# MALDI-TOF Mass Spectroscopy Applications in Clinical Microbiology

**DOI:** 10.1155/2021/9928238

**Published:** 2021-05-07

**Authors:** Maryam Alizadeh, Leila Yousefi, Farzaneh Pakdel, Reza Ghotaslou, Mohammad Ahangarzadeh Rezaee, Ehsaneh Khodadadi, Mahin Ahangar Oskouei, Mohammad Hossein Soroush Barhaghi, Hossein Samadi Kafil

**Affiliations:** ^1^Student Research Committee, Faculty of Medicine, Tabriz University of Medical Sciences, Tabriz, Iran; ^2^Research Center for Pharmaceutical Nanotechnology, Faculty of Medicine, Tabriz University of Medical Sciences, Tabriz, Iran; ^3^Immunology Research Center, Faculty of Medicine, Tabriz University of Medical Sciences, Tabriz, Iran; ^4^Drug Applied Research Center, Faculty of Medicine, Tabriz University of Medical Sciences, Tabriz, Iran; ^5^Stem Cell Research Center, Faculty of Medicine, Tabriz University of Medical Sciences, Tabriz, Iran; ^6^Pharmaceutical Analysis Research Center, Tabriz University of Medical Sciences, Tabriz, Iran

## Abstract

There is a range of proteomics methods to spot and analyze bacterial protein contents such as liquid chromatography-mass spectrometry (LC-MS), two-dimensional gel electrophoresis, and matrix-assisted laser desorption/ionization mass spectrometry (MALDI-TOF MS), which give comprehensive information about the microorganisms that may be helpful within the diagnosis and coverings of infections. Microorganism identification by mass spectrometry is predicted on identifying a characteristic spectrum of every species so matched with an outsized database within the instrument. MALDI-TOF MS is one of the diagnostic methods, which is a straightforward, quick, and precise technique, and is employed in microbial diagnostic laboratories these days and may replace other diagnostic methods. This method identifies various microorganisms such as bacteria, fungi, parasites, and viruses, which supply comprehensive information. One of the MALDI-TOF MS's crucial applications is bacteriology, which helps identify bacterial species, identify toxins, and study bacterial antibiotic resistance. By knowing these cases, we will act more effectively against bacterial infections.

## 1. Introduction

Bacterial pathogens are among the factors that threaten global health and can grow in humans [1], animals [2], and plants [3] and cause disease in their hosts under certain conditions, including the acquisition of virulence factors [4, 5]. These microorganisms have different macromolecules in their structure, including genome [6], proteins [7, 8], polysaccharides [9], and phospholipids [10], which various techniques and methods can study.

To study bacterial macromolecules, there are various methods, including genomics and proteomics. The genomics methods give us broad information about the bacteria's genome, and proteomics methods help us identify multiple bacterial proteins [11, 12]. The proteomics methods are liquid chromatography-mass spectrometry (LC-MS) [13–15], two-dimensional gel electrophoresis [16–19], and matrix-assisted laser desorption/ionization mass spectrometry (MALDI-TOF MS) [20–22], which are used for studying bacterial protein contents and give comprehensive information about the cell and can be helpful in the identification of the bacteria, and so treatment of the infections is performed well timed by creating effective drugs and vaccines [23].

MALDI-TOF MS is one of the most potent proteomics methods that can detect various microorganisms such as bacteria [24], viruses [25–27], fungi [28–30], and parasites [31–33] and also can provide us precise, quick, easy, and inexpensive comprehensive information about microorganisms [34]. This study aimed to comprehensively investigate the applications of MALDI-TOF in bacteriological studies and the advantages of this method over other methods of laboratory diagnosis of infections.

## 2. The General Applications of MALDI-TOF MS

There are various methods for identifying different cells; the study of cell proteins is one of those methods, which has been used in diagnostic laboratories for many years [35–37]. MALDI-TOF MS technique is a successful method of detecting microorganisms that have been widely used in microbiological laboratories in recent years [38–40].

**Figure 1:**
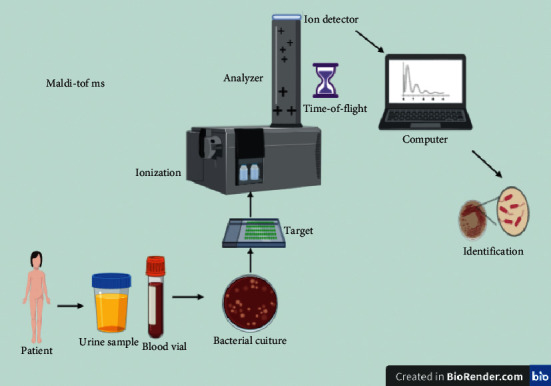
MALDI-TOF MS that includes an ion source to generate ions from the sample, one or more mass analyzers to separate ions based on mass-to-charge ratio, a detector to record the number of ions removed from the analyzer, and a computer to process data and create spectrum and control the device through the feedback, which can use these data to get comprehensive information about the microorganisms.

MALDI-TOF MS is a device that generates ions from a sample and then separates the generated ions based on the mass-to-charge ratio in the gas phase [41]. Today, mass spectrometry is a susceptible method for the structural study of biomolecules [42]. At present, each mass spectrometer includes an ion source to generate ions from the sample, one or more mass analyzers to separate ions based on mass-to-charge ratio, a detector to record the number of ions removed from the analyzer, and a computer to process data and create spectrum and control the device through the feedback [43], which can use these data to get comprehensive information about the microorganisms (Figure 1).

An essential feature of MALDI-TOF MS is that in addition to detecting bacteria, it can be used to detect microbial toxins [44], study antibiotic resistance [45–47], detect viruses [48, 49], parasites [50, 51], fungi [52, 53], and study of human [54–56] and plant cells [57, 58], which makes us more familiar with the creatures in our environment.

Essential applications of MALDI-TOF MS in virology include identifying various mutations in viruses and identifying different microorganisms' strains, which might help the rapid and accurate diagnosis of viruses [49]. For example, in the rapid detection of respiratory viruses including influenza A, influenza B, respiratory syncytial virus (RSV), parainfluenza viruses, adenovirus, and rhinovirus, the MALDI-TOF MS method is used, which has increased the speed and accuracy in the diagnosis of these viruses by detection of their protein structure such as nucleoprotein (NP), haemagglutinin (HA), neuraminidase (NA), matrix protein (M1), and a nonstructural protein (NS1) in the influenza A, which is one of the advantages of this method compared to other traditional diagnostic procedures, such as serology, cell culture, and real-time reverse transcription-polymerase chain reaction (rRT-PCR) [59], which by the rapid diagnosis of infection can help in the immediate initiation of treatment and surviving the patients.

Another application of MALDI-TOF MS is in mycology; it effectively identifies different fungal species, identifies subspecies, compares differences between other fungi, and identifies fungal resistance antifungal drugs [60]. For example, in the study by Antonietta Vella et al. [61], they used MALDI-TOF MS to evaluate the antifungal resistance of *Candida glabrata* against anidulafungin and fluconazole, and this technique shows the antifungal resistance of these fungi rapidly.

MALDI-TOF MS also is used in parasitology, where it is used to detect various parasites such as *Leishmania* [62], *Cryptosporidium parvum* [63], *Giardia lamblia* [64], and *Entamoeba histolytica* [65], which provides us with comprehensive information about them.

## 3. Identification of Bacteria

Until recently, only phenotypic and genotypic methods were used to detect bacteria in bacteriological laboratories [66], and by the advancement of science, other techniques were used [67], such as MALDI-TOF MS, which is based on the study of bacterial proteins, which is an easy, fast, and accurate method that can replace other diagnostic procedures [68]. This method makes mass spectral for each microorganism unique as a fingerprint, identifying different bacterial genus and subspecies [34].

Bacteria use protein for various structural and functional purposes. For example, they use protein to build many parts such as cell envelope [69], flagella [70], secretory systems [71], enzymes [72], and biofilm [73, 74], and MALDI-TOF MS helps detecting various bacteria by detecting these proteinaceous parts.

MALDI-TOF technique has been used in bacteriology to study different types of bacteria such as Gram-positive [75], Gram-negative [76], mycobacteria [77], and anaerobic [78], which give complete and comprehensive information about those microorganisms, useful in the fast diagnosis of bacterial infections. For example, in the study of Sun et al. [79], they used MALDI-TOF MS to identify species of *Lactobacillus plantarum,* a Gram-positive bacterium, and they identified 34 proteins as the biomarker proteins and used these proteins for identifying species of bacterium in the various cultures. In another study by Friedrichs et al. [80], MALDI-TOF MS was used to identify 99 species of clinical *Streptococci,* a Gram-positive bacterium; they identified 71 *Streptococcus mitis,* 23 *Streptococcus anginosus,* and five *Streptococcus salivarius*. Another study carried out on *mycobacteria* by Hettick et al. [81] used MALDI-TOF MS to identify various mycobacteria and acid-fast bacteria, such as *Mycobacterium tuberculosis*, *Mycobacterium intracellulare*, *Mycobacterium avium*, *Mycobacterium bovis*, *Mycobacterium kansasii*, and *Mycobacterium fortuitum.* Due to the development of tuberculosis by the *M. tuberculosis*, which is one of the most dangerous global infections and health problems, rapid and accurate diagnosis of this bacterium is very important, and the use of the MALDI-TOF MS technique can be helpful in this operation [82].

Another vital application of MALDI-TOF MS is identifying anaerobic bacteria that face many difficulties due to their hard growing on solid media, the ineffectiveness of routine biochemical tests for their identification, and the need for specific environmental conditions that develop the need for new and effective diagnostic procedures [83]. For example, the study of Eigner et al. [84] is on more than 1000 bacteria isolated from laboratories, since the detection of 95.2% of isolation was correct, and shows this method's effectiveness for detecting anaerobe bacteria.

## 4. Identification of Bacterial Toxin

Different microorganisms such as bacteria produce proteins called toxins to increase their virulence [85]. The toxin is a part of the bacterial structure called endotoxin [86] or secretes out of the cell called exotoxin [87]. Many bacteria form toxins that can be identified by different MALDI-TOF MS methods, which help in their detection [88]. For example, *Staphylococcus aureus* threatens people's health by causing dangerous infections by producing a toxin called Panton–Valentine leukocidin (PVL); fast and accurate detection of this toxin is crucial to save the life of people [89]. In the study of Bittar et al. [90], they used MALDI-TOF MS for detecting PVL for identification of 81 *S. aureus* isolated from patients of a hospital, and they were able to identify the isolates effective, fast, and efficiently by using this method.

Another study by Ranasinghe and Akhurst [91] was performed to study crystal toxin of *Bacillus thuringiensis* by MALDI-TOF MS to detect these bacteria and shows that this method can identify the toxin rapidly and sensitively that indicates the effectiveness of this method in rapid identification of microbial toxins for quick control of the infection.

## 5. Study of Antibiotic Resistance

Antibiotic resistance is the ability of the microorganisms to resist medications that are used to destroy them. This resistance can be caused by excessive consumption of antibiotics and a mutation in microbes that have caused this phenomenon to become a global health threat [92].

To resist antibiotics, the bacteria attempt to perform various actions, including constructing specific structures called efflux pumps, which drive the antibiotic out of the bacteria [89] and make enzymes that can destroy antibiotics [93]. For fast, accurate, and effective detection of antibiotic-resistant bacteria, new methods must identify the factors that make the bacteria resist antibiotics. MALDI-TOF MS is one of these methods that can locate efflux pumps and enzymes made up of protein [94].

Rapid identification of some bacteria is essential and plays a vital role in preventing transmission to other people, and delays in identifying them can lead to countless deaths, which indicate the need for rapid diagnostic methods such as MALDI-TOF MS. Among these bacteria, we can mention methicillin-resistant *Staphylococcus aureus* (MRSA), one of the main threats to public health. For example, in a study by Edwards-Jones et al. [95], they used MALDI-TOF MS to detect MRSA. Its fast identification is vital for appropriate therapeutic actions and timely interposition for controlling the infection.

## 6. Advantages and Disadvantages of MALDI-TOF MS

MALDI-TOF approaches effectively discover viruses in a wide range of biological specimens, and the concordance rate between MALDI-TOF and other standard-based methods is high [96]. PCR-based identification methods have some restrictions such as a lot longer turn-around time than MS, reagent and labor expenses, and some practical issues [97]. On the other hand, MALDI-TOF modern technology can likewise be applied to recognize infections in coinfected tests. These approaches can synchronize the discovery of several pathogens in a solitary essay. They can then inhibit misdiagnosis and delays in treatment without rising costs or adding a new action to the procedure [98].

RT-PCR procedures might have repressive fragments and contamination problems; hence, different sample preparation, amplification, and analysis are required [99]. Additionally, nucleic acid-based methods are costly and lengthy and appear in many cases much less convenient than MS for regular laboratory recognition [100, 101]. For MALDI-TOF, there needs to be substantial development in closing the upfront of PCR processing to consist of DNA extraction and the PCR procedure itself. MALDI-TOF MS approaches likewise enable large-scale research studies for fresh samples and archival samples from numerous biological specimens [102].

One of the significant weaknesses of MALDI-TOF is that the database restricts the identification [50]. The mass profile is used as a mass spectrum to compare well-characterized microorganisms in a database [103]. The range generally consists of specific peaks, so that with an extensive collection of spectra, the recognition may be carried out by applying bioinformatics [104].

## 7. Future Perspectives

The application of MS to molecular diagnostics has become a highly active part of the study with considerable ramifications for medication and public health. One of the most sophisticated advancements has been created by precise matching of molecular methods with MALDI-TOF. MS as a discovery system is most matched for the recognition of complicated genetic markers without invoking sequences [105]. It can be anticipated that the future growth of MS-based molecular diagnostics will be connected to unique techniques of extracting scientifically and epidemiologically relevant info such as disorder intensity, medicine resistance, vaccination retreat, and transmission from the genetic markers applying mainly developed computational and mathematical versions [106]. An essential feature of the MS innovation will be the possibility of its application to the quick discovery of microbes triggering hospital infection [107]. If combined with proper fast and delicate modern technologies for the medical diagnosis of avoidable diseases, MS can also influence quality control of sterile blood items and food security [108]. Assimilation of molecular and computational strategies with MS needs to generate analysis assays for wide, regular public health and medical practice applications.

## 8. Conclusion

Rapid diagnosis of infection makes the control of it more straightforward, vital for protecting the community's health. There are various methods for rapid diagnosis of infections. One of them is MALDI-TOF MS, which is a fast, easy, and accurate method. Detecting bacterial proteinaceous agents helps us diagnose disorders quickly, which leads to more rapid treatment of the patient.

## Data Availability

The data used to support the findings of this study are available from the corresponding author upon request.
